# Modifications of the readiness assessment for pragmatic trials tool for appropriate use with Indigenous populations

**DOI:** 10.1186/s12874-024-02244-z

**Published:** 2024-05-31

**Authors:** Joanna Hikaka, Ellen M. McCreedy, Eric Jutkowitz, Ellen P. McCarthy, Rosa R. Baier

**Affiliations:** 1 Department of General Practice and Primary Health Care, University of Auckland, Auckland, New Zealand; 2 Center for Long-Term Care Quality & Innovation, Brown University School of Public Health, Providence, RI USA; 3 Department of Health Services, Policy & Practice, Brown University School of Public Health, Providence, RI USA; 4 Providence Veterans Affairs Medical Center, Providence VA, RI USA; 5 Hinda and Arthur Marcus Institute for Aging Research, Hebrew SeniorLife, Boston, MA USA; 6 Division of Gerontology, Department of Medicine, Beth Israel Deaconess Medical Center, Harvard Medical School, Boston, MA USA

**Keywords:** Implementation science, Health services research, Indigenous health, Data sovereignty, Equity

## Abstract

**Background:**

Inequities in health access and outcomes exist between Indigenous and non-Indigenous populations. Embedded pragmatic randomized, controlled trials (ePCTs) can test the real-world effectiveness of health care interventions. Assessing readiness for ePCT, with tools such as the Readiness Assessment for Pragmatic Trials (RAPT) model, is an important component. Although equity must be explicitly incorporated in the design, testing, and widespread implementation of any health care intervention to achieve equity, RAPT does not explicitly consider equity. This study aimed to identify adaptions necessary for the application of the ‘Readiness Assessment for Pragmatic Trials’ (RAPT) tool in embedded pragmatic randomized, controlled trials (ePCTs) with Indigenous communities.

**Methods:**

We surveyed and interviewed participants (researchers with experience in research involving Indigenous communities) over three phases (July-December 2022) in this mixed-methods study to explore the appropriateness and recommended adaptions of current RAPT domains and to identify new domains that would be appropriate to include. We thematically analyzed responses and used an iterative process to modify RAPT.

**Results:**

The 21 participants identified that RAPT needed to be modified to strengthen readiness assessment in Indigenous research. In addition, five new domains were proposed to support Indigenous communities’ power within the research processes: Indigenous Data Sovereignty; Acceptability – Indigenous Communities; Risk of Research; Research Team Experience; Established Partnership). We propose a modified tool, RAPT-Indigenous (RAPT-I) for use in research with Indigenous communities to increase the robustness and cultural appropriateness of readiness assessment for ePCT. In addition to producing a tool for use, it outlines a methodological approach to adopting research tools for use in and with Indigenous communities by drawing on the experience of researchers who are part of, and/or working with, Indigenous communities to undertake interventional research, as well as those with expertise in health equity, implementation science, and public health.

**Conclusion:**

RAPT-I has the potential to provide a useful framework for readiness assessment prior to ePCT in Indigenous communities. RAPT-I also has potential use by bodies charged with critically reviewing proposed pragmatic research including funding and ethics review boards.

**Supplementary Information:**

The online version contains supplementary material available at 10.1186/s12874-024-02244-z.

## Background

The World Health Organization defines health equity as ‘the absence of unfair, avoidable or remediable differences among groups of people’ and states ‘health equity is achieved when everyone can attain their full potential for health and wellbeing’ [1]. Healthcare access and outcomes differ between Indigenous and non-Indigenous populations across the globe, are unfair and unjust, and are therefore defined as health inequities [2]. These inequities are mediated by colonization and structural racism, which reduce Indigenous peoples’ access to the wider determinants of health, such as education, employment, and healthcare access, further affecting the barriers and enablers of high-quality health care [3]. To achieve Indigenous health equity [4 (p2)] equity must be explicitly incorporated in the design, testing, and widespread implementation of any intervention [5–9]. In practice, this requires researchers to work together with Indigenous communities to understand local contexts and support the achievement of equity by involving Indigenous people as leaders in research, understanding Indigenous priorities, aspirations, and appropriate measures of success [5, 6, 10]. A recent publication of an equity-focused implementation framework provides practical guidance on how to incorporate equity [11]. The framework is founded on Indigenous rights as set out in New Zealand’s (NZ’s) founding legislative document and includes steps such as defining resources required for equitable implementation [11].

Centuries of colonial research and inquiry involving subjugation of Indigenous peoples by powerful ‘others’ provides a lineage to contemporary research practices which further exclude and marginalize Indigenous populations [7]. This exclusion and marginalization is seen in health intervention research. Indigenous populations may be ‘unseen’ through non-reporting of participants’ ethnicity, or under-represented through low Indigenous recruitment [12]. The design of the trial, outcome measures, or the intervention itself, may be culturally inappropriate or not reflect Indigenous priorities [10, 13]. Findings may also be inappropriately framed to focus on individual or cultural deficits rather than service or systematic factors contributing to differences in outcomes between Indigenous and non-Indigenous populations [14, 15]. As a result, interventional research that demonstrates benefit in predominately White populations may not be effective, feasible, or acceptable in other cultural settings and research tools developed in non-Indigenous settings have the potential to widen inequities [16] and lead to unethical research practices in Indigenous populations [17].

Explicitly designing for equitable health access and outcomes at the outset facilitates pro-equity research. Indigenous pro-equity research may be supported using embedded pragmatic randomized, controlled trials (ePCTs). ePCTs are effectiveness trials that reflect real-world considerations [18], including ensuring research is appropriate to the targeted communities and settings [7, 19]. Further preparatory work is likely required to prepare interventions shown to be effective in predominately non-Indigenous populations for ePCTs in Indigenous populations. Previous Indigenous health intervention research undertaken in Australia, Canada, the United States (US) and NZ has identified processes for investigating how to co-design, implement and evaluate interventions in Indigenous settings [20], adapting interventions prior to ePCT [21], how to ensure low resource environments are ready to implement an intervention within a ePCT [22], as well as targeting specific research processes, such as recruitment [23].

An intervention must be sufficiently ‘ready’ for an ePCT to ensure it will be feasible to conduct and possible to draw appropriate conclusions from the findings [24]. The Readiness Assessment for Pragmatic Trials (RAPT) model is an implementation science tool to help researchers qualitatively assess an intervention’s ‘readiness’ (low to high) in the context the intervention’s current state and likelihood of intervention adoption if proven effective in ePCT [25]. There are nine domains with accompanying questions and scoring criteria: [25]

### Implementation protocol

Is there an implementation protocol that is sufficiently detailed to enable replication?

### Evidence

What is the extent of evidence to support intervention efficacy?

### Risk

Is the safety of the intervention known?

### Feasibility

To what extent can the intervention be implemented within the current environment?

### Measurement

To what extent can the intervention effectiveness be measured, ideally using pragmatic outcome measures?

### Cost

Is the intervention likely to be economically viable?

### Acceptability

How likely is it that providers will adopt the intervention?

### Alignment

To what extent is the intervention in alignment with stakeholders’ priorities?

### Impact

How likely is it that the results for the ePCT will inform clinical practice and/or policy?

RAPT’s readiness domains were defined based on discussion amongst experts at a US National Institute on Aging workshop. However, the resulting model does not explicitly include health equity [26] and has not been applied to pro-equity Indigenous health intervention research. If adapted to include Indigenous equity considerations, RAPT may inform such efforts. This study aimed to identify adaptions necessary for RAPT’s application to ePCTs with Indigenous communities.

## Methods

### Study design

This mixed-methods study used an online questionnaire and semi-structured interviews. This study was approved by the Auckland Health Research Ethics Committee (AH24242).

This research was led by JH, an Indigenous health services researcher from NZ with experience in Indigenous research methodology and qualitative research, including inductive thematic analysis in Indigenous research underpinned by Indigenous theory. She was working at a university in the US at the time this research was undertaken and worked in collaboration with the rest of the research team who are the lead authors of the RAPT model. Our research team had expertise in qualitative research, co-design and co-creation, public health, health equity, survey methodology, quality improvement, and clinical care in older adult settings. The researchers recognise the right of Indigenous peoples, and the right of people living with dementia, to experience equitable health outcomes.

### Recruitment and consent

#### Eligibility

Participants were eligible if they were 18 years or older and had been involved as a researcher (self-identified, no formal qualifications required) in research relating to non-pharmacological dementia care interventions in Indigenous communities in NZ (Māori) or the United States (US; American Indian, Native Alaskan, and Kānaka Maoli/Native Hawaiian peoples). We focused on dementia interventions because RAPT, although since applied more broadly [27], was initially developed to assess dementia interventions [25] and because this work was partially conducted in partnership with the US National Institute on Aging (NIA) IMPACT Collaboratory, which focuses on dementia interventions. NZ and the US were the countries of interest as the lead author is an Indigenous researcher from NZ and was a visiting scholar, collaborating with the US authors of RAPT.

#### Recruitment

We first conducted a literature search to identify peer-reviewed publications relating to non-pharmacological dementia interventions (any study design) that included Indigenous populations in the US or NZ and were published from 2011 to 2022. We then emailed invitations to all identified authors for whom we could obtain email addresses (*n* = 77). We also emailed invitations to directors of three Indigenous ageing research centers and the International Indigenous Dementia Research Network. We used snowball techniques to identify additional potential study participants [28]. Participants provided informed consent using an online form immediately prior to completing the online questionnaire.

### Questionnaire development and data collection

We surveyed participants in July and August 2022. We provided brief introductory material regarding RAPT. We then asked participants to complete the questionnaire (Supplementary material). We collected all data using Qualtrics® (Seattle, Washington US).

#### Demographics and research experience

The questionnaire captured respondents’ demographics, including self-identified ethnicity and research experience.

#### RAPT domain questionnaire

We asked participants first to reflect on their research experiences, then to rate each RAPT domain’s appropriateness for interventional research with Indigenous communities using a 4-point Likert scale (inappropriate, slightly inappropriate, slightly appropriate, appropriate). We also asked participants to indicate whether ‘to adequately incorporate health equity’ a domain needed any modifications or should be removed. If they advised modifications, we asked for specific suggestions.

### Semi-structured interviews and consensus building

After modifying the existing RAPT domains and adding new domains based on participants’ questionnaire responses, we drafted a modified RAPT, termed the RAPT-Indigenous (RAPT-I). We conducted a semi-structured in-depth interview with respondents to the online questionnaire component (‘respondents’) who assented to participate in follow-up interviews (November-December 2022). Questionnaire respondents were invited to participate rather than new participants to continue development and refinement of domains, similar to the approach taken in a Delphi consensus approach [29] and to methods used in other similar implementation science research [22]. The lead author (JH) conducted all interviews using Zoom™ (San Jose, California US) and transcribed the interviews. We provided participants (interviewees) with the draft RAPT-I via email at the time of scheduling the interview, encouraging them to review draft RAPT-I ahead of the interview. During interviews, we explored interviewees perspectives about RAPT-I; any guidance that should accompany the tool; whether ePCTs in Indigenous populations should proceed with low readiness in various domains; and the modified tool’s utility with marginalized populations other than Indigenous communities. Interviewees could request a recording of their interview within two weeks of the interview. An iterative process was used to make further modifications to the draft RAPT-I based on interview responses.The lead researcher sent a second RAPT-I draft to all interviewees and invited them to review and suggest additional modifications prior to RAPT-I’s finalisation. Interviewees provided further feedback either in written form or via a video conference where notes were taken by the lead researcher.

### Data analysis

**Fig. 1:**
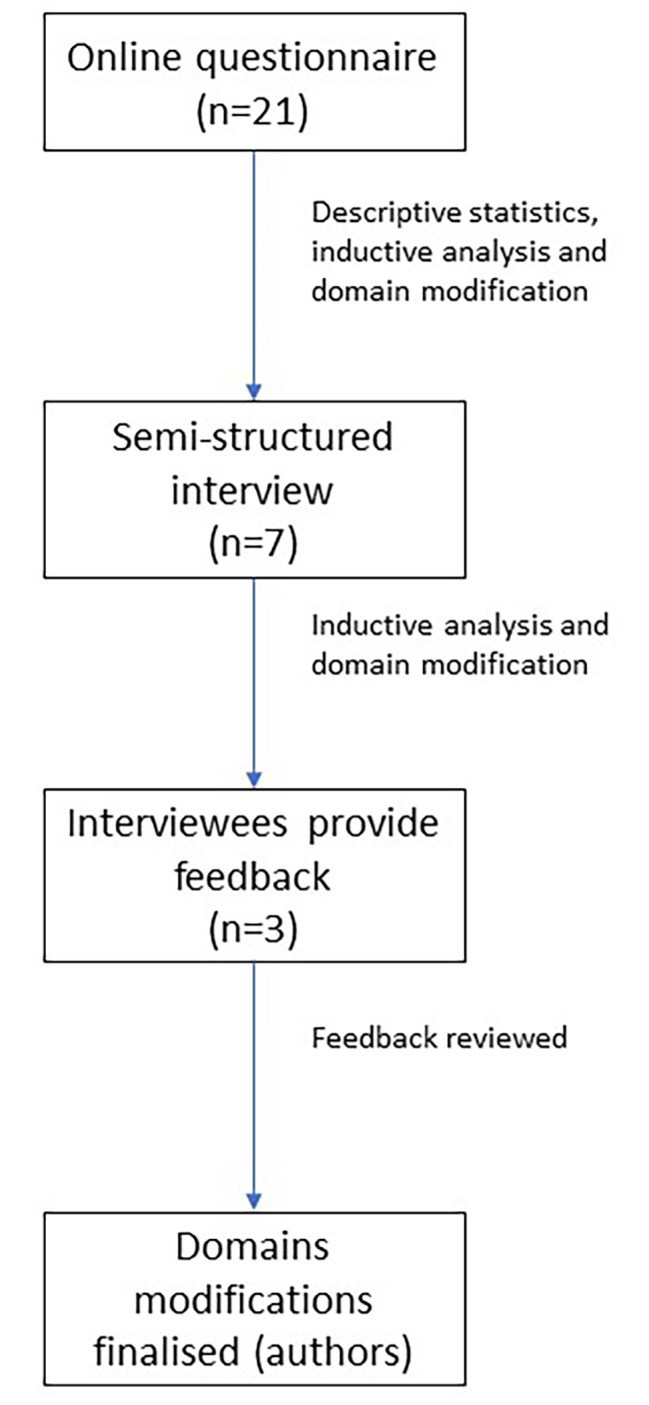
Participant flow through study

We used Microsoft Excel® (Seattle, Washington US) to characterize participants using descriptive statistics. We calculated the percentage of participants who selected each Likert response when asked about each domain’s appropriateness and need for modification. The lead researcher used the current domains as a framework to group qualitative feedback from the questionnaire and interviews that related to each of the existing domains [30] and used general inductive analysis to generate new domains from free-text questionnaire responses and interview transcripts to develop new domains (Fig. 1). This preliminary analysis was presented to all other authors for discussion and review, with raw data being supplied as required during discussions. A general inductive approach was chosen as this method aligns with our intent to condense and summarize extensive and varied raw data and to develop a model [31], in this case a modification of RAPT. We included quotes from respondents (‘R’) in the results. We did not undertake any subgroup analysis. For each of the stages that involved iterative changes to draft versions of RAPT-I, the lead researcher made initial changes which were then discussed with all other authors for consensus building and finalization of draft versions. The lead author undertook the final iterative review process which produced a third draft that was finalized, through consultation and discussion with the full research team, for presentation in this paper.

### Sample size

We targeted 30 participants to reach saturation of responses to qualitative questionnaire questions. We aimed for approximately 15 participants from each country and at least 10 who self-identified as Indigenous.

## Results

We emailed questionnaire invitations to 77 people and 21 (27·3%) responded. Research experience ranged from 5 to 40 years (median 20 years); experience focused on older adult/dementia research, 3–40 years (median: 10 years); and with Indigenous research 4–18 years (median: 6). Two-thirds of participants were from NZ (*n* = 14, 66·7%). About half identified as Indigenous (*n* = 10, 47·6%) or White (*n* = 9, 42·9%); the remainder, non-Indigenous ethnic/racial minorities (*n* = 2, 9·5%).

Seven (33·3%) questionnaire respondents participated in follow-up interviews, which lasted 26–30 min (median: 28 min). Research experience ranged from 5 to 28 years (median: 14 years). Four interviewees (57·1%) were from NZ; all but one (*n* = 6, 85·7%) identified as Indigenous; one an ethnic minority; and three were female (42·9%). Following the interviews, three interviewees (42·9%) reviewed the draft RAPT-I. Saturation of ideas in response to qualitative survey questions was achieved. Saturation of interviewee responses was not sought although saturation was largely achieved after the fifth interview. Interviewees suggested further changes to the first draft RAPT-I which focused on the clarification of domain and scoring wording to convey intended meaning, highlighting the importance of Indigenous partnership, and the value of accompanying guidance to support the use of RAPT-I.

All nine domains were assessed as being appropriate or slightly appropriate by most participants (Table 1); however, most participants (90·5%) indicated that some modifications were needed to increase appropriateness for use with Indigenous populations. A greater proportion of respondents would use a modified version of RAPT (*n* = 15, 71·4%) vs. the original (*n* = 8, 38·1%).

**Table 1:** Appropriateness and recommendations for modification of RAPT domains for use with Indigenous communities (*N* = 21)

		Appropriateness of domain						
**RAPT Domain**		Appropriate (%)		Slightly appropriate (%)		Slightly inappropriate (%)		Inappropriate (%)
**Implementation protocol (** ***n*** **= 21)** *Is the protocol sufficiently detailed to be replicated?*		15 (71·4)		4 (19·0)		2 (9·5)		0 (0·0)
**Evidence (** ***n*** **= 19)** *To what extent does the evidence base support the intervention’s efficacy?*		8 (42·1)		8 (42·1)		2 (10·5)		1 (5·3)
**Risk (** ***n*** **= 19)** *Is it known how safe the intervention is?*		10 (52·6)		4 (21·1)		3 (15·8)		2 (10·5)
**Feasibility (** ***n*** **= 19)** *To what extent can the intervention be implemented under existing conditions?*		13 (68·4)		3 (15·8)		3 (15·8)		0 (0·0)
**Measurement (** ***n*** **= 19)** *To what extent can the intervention’s outcomes be captured?*		12 (63·2)		5 (26·3)		2 (10·5)		0 (0·0)
**Cost (** ***n*** **= 19)** *How likely is the intervention to be economically viable?*		12 (63·2)		3 (15·8)		4 (21·1)		0 (0·0)
**Acceptability (** ***n*** **= 18)** *How willing are providers likely to be to adopt the intervention?*		11 (61·1)		3 (16·7)		4 (22·2)		0 (0·0)
**Alignment (** ***n*** **= 18)** *To what extent does the intervention align with external stakeholders’ priorities?*		10 (55·6)		3 (16·7)		4 (22·2)		1 (5·6)
**Impact (** ***n*** **= 18)** *How useful will the intervention’s results be?*		12 (57·1)		3 (16·7)		2 (11·1)		1 (5·6)
	**Level of modification**							
**RAPT Domain**	Unchanged (%)		Additions (%)		Aspects Removed (%)		Rewritten (%)	Removed (%)
Implementation protocol (*n* = 21)	5 (23·8)		14 (66·7)		0 (0·0)		2 (9·5)	0 (0·0)
Evidence (*n* = 19)	4 (21·1)		10 (52·6)		1 (5·3)		4 (21·1)	0 (0·0)
Risk (*n* = 19)	7 (36·8)		7 (36·8)		0 (0·0)		5 (26·3)	0 (0·0)
Feasibility (*n* = 19)	6 (31·6)		11 (57·9)		0 (0·0)		2 (10·5)	0 (0·0)
Measurement (*n* = 19)	9 (47·4)		8 (42·1)		0 (0·0)		2 (10·5)	0 (0·0)
Cost (*n* = 19)	7 (36·8)		6 (31·6)		1 (5·3)		4 (21·1)	1 (5·3)
Acceptability (*n* = 18)	6 (33·3)		8 (44·4)		0 (0·0)		3 (16·7)	1 (5·3)
Alignment (*n* = 18)	7 (38·9)		8 (44·4)		0 (0·0)		2 (11·1)	1 (5·6)
Impact (*n* = 18)	6 (28·6)		8 (44·4)		0 (0·0)		4 (22·2)	0 (0·0)

### General summary of questionnaire responses to existing domains

Although respondents felt many domains were general enough to be appropriate, most recommended including explicit guidance regarding the intent to achieve Indigenous health equity and to minimise potential risks associated with the intervention or research process. Many felt such guidance would promote culturally-safe interventions and research practices, help researchers to identify areas to strengthen before an ePCT, and even provide a framework for critical review by funders and ethics boards.

*The goal of [using a tool such as RAPT] is that health equity becomes part and parcel of how we do high quality research.* (R10, US, non-Indigenous ethnic minority)

At the same time, they expressed any guidance provided needed to support meaningful assessment rather than performative assessment that did not change approaches to research.

*The question is, will it become another tick box exercise?* (R3, NZ, Indigenous)

#### Implementation protocol

All respondents felt that it was appropriate to have an implementation protocol that considered equity through all aspects of implementation.

*The parameters and criterion of health equity should be demonstrated.* (R15, NZ, Indigenous)

However, many noted there were likely to be aspects of pragmatic research with Indigenous communities that could not be protocolised. Others noted that even if a protocol *enabled* replicability, replicating an intervention tested in one Indigenous community in another Indigenous community may be inappropriate.

*There is a need to recognize the flexibility necessary for Indigenous research, I believe there is an option between partially documented and fully documented, for flexible documentation that is mostly (or partially) documented, that is revised during the research journey.* (R4, NZ, Indigenous)

#### Evidence

Many respondents deemed Evidence essential; however, most recommended requiring evidence with the targeted Indigenous community specifically. Several questioned the need for efficacy evidence from randomized-controlled trials, which may not be available for Indigenous communities.

*The ideal is to have prior evidence, however there may not be prior evidence for Indigenous populations. Sometimes a number of less rigorous methods is good enough evidence for the intervention to be tested.* (R4, NZ, Indigenous)

Some respondents also felt that it was important to modify Evidence to include evidence of access- and equity-related outcomes. One noted that one purpose for conducting research with Indigenous communities may be that interventions efficacious in other populations either do not achieve equity or worsen inequities in Indigenous populations.

*In many instances of health equity research, there may not be any existing efficacy studies. I mean, a large part of the drivers of inequity are that interventions are NOT fit for purpose and it is precisely because of this that new interventions are being proposed and researched!”* (R3, NZ, Indigenous).

#### Feasibility

Most participants agreed that Feasibility was important and that understanding feasibility specifically in the targeted Indigenous community was crucial, as it may differ across populations. For example, some participants shared that it may not be possible to adequately implement a new intervention with existing resources in already under-resourced communities or populations.

*[Feasibility] tends to be neglected as work is moved into communities.* (R9, US, non-Indigenous)

Several participants felt that additional support (e.g., human and financial resourcing) may be required to investigate a new intervention and that such needs should not be reason to withhold research opportunities from communities already experiencing inequitable resourcing.

#### Measurement

Many participants felt that pragmatic outcome data collection could be beneficial, but potentially unachievable for Indigenous interventions, for example if structural inequities impacted the availability and use of electronic health records systems.

*Rural indigenous communities do not have outcomes “routinely captured” due to lack of health care / poor health care services.* (R7, US, Indigenous)

One participant questioned the ability of electronic systems to accurately capture measures, noting that ethnic minority populations are routinely misclassified and undercounted in NZ national data sets. Another suggested that measurement readiness could be expanded to include two items: one focused on exploring electronic data collection; the other, on using easily collected and entered hand-written data collection.

#### Cost

Most participants deemed the economic viability important for sustainability and evidence-based resource allocation. However, they felt that expertise in cost-benefit analysis *in the Indigenous communities of interest* would be important to appropriately account for economic costs or benefits particular to the community of interest and to consider the wider influences and impacts of inequitable resourcing in health service/system infrastructure and in the social determinants of health.

*To achieve equity the costs are often higher in these populations to achieve the same level of intervention/outcome.* (R6, NZ, non-Indigenous)

Equally, some participants described the need to take a broad approach to assessing benefits through an Indigenous lens, e.g., improvement in spiritual wellbeing or social connectedness.

Importantly, some participants related Cost to Evidence, noting lack of evidence in Indigenous communities would affect cost-benefit analysis calculations or considerations. Several felt that lack of cost data or low readiness should not prevent investigation of potentially beneficial interventions in “understudied, underserved, and minoritized groups”.

#### Impact

Some participants were unclear about the distinctions among Acceptability, Alignment, and Impact and suggested adding wording to clarify differences. As currently framed, Acceptability and Alignment domains focus on the existing relevance to internal and external stakeholders, whereas Impact domain focuses on the potential value of future ePCT findings [12]. Participants felt Impact aligned with Indigenous values by appropriately focusing on the potential for translation into practice, but that the domain needed to focus on benefit for Indigenous communities and inform or relate to *equitable* clinical care and policy.

*Impact should be Indigenous focused. A focus group would be better able to define how this would look when considering what qualifies as meaningful “impact”.”* (R7, US, Indigenous).

Further respondent quotes are shown in Table 2.

**Table 2:** Participant perceptions of domain appropriateness and modifications

Domain	Illustrative Quote
Implementation protocol	*“This domain in a generic way could be considered to cover everything that is needed (e.g. is there a protocol or not, and how well documented), but provide little depth/detail as to what this actually looks like.”* R6, NZ, non-Indigenous *“… protocols need to be custom for the community in order to be effective; sufficient detail for Indigenous communities would be better served if the intervention is written in non-scientific jargon and can be conducted by minimally trained indigenous professionals.”* R7, US, Indigenous
Evidence	*“This is key -- there has to be some indication of evidence to the population in question (here, indigenous groups). Perhaps a lower level of “readiness” may be acceptable (some vs. none) but there has to be some indication that the intervention works prior to deploying it as a PCT.”* R10, US, non-Indigenous ethnic minority
Risk	*“This domain is mixing up whether risks are known or unknown, with the level of risk. It also does not recognise “cultural risk” or communal/community risk and only focuses on individual daily life risk.”* R4, NZ, Indigenous *“Harm in Indigenous research can be from aspects not previously thought about in mainstream research; thus harm has been considered from all angles and is likely to be minimal may be more appropriate.”* R12, NZ, non-Indigenous
Feasibility	*“Could there be follow up questions to focus on this issue? If the issue is with infrastructure or funding then that is at a different level to if the issue was with staffing.”* R2, NZ, non-Indigenous *“From a strengths-based perspective, it will be helpful to add details focused on community strengths and resources that currently exist (less deficit-based that will make communities feel they are unprepared or not able to support intervention).”* R5, US, Indigenous
Measurement	*“Outcomes are important to capture and if lots more time and effort are needed, that is less desirable.”* R12, NZ, non-Indigenous *“Need to acknowledge that existing outcome measures may not be designed to capture equity issues or measures important to specific cultural groups.”* R3, NZ, Indigenous
Cost	*“What are the intangible outcomes that haven’t been considered?”* R19, NZ, Indigenous *“Cost-benefit is no doubt important but as noted in earlier answers, a lot of health equity research is needed because the current system (cost-effective or otherwise) is not fit for purpose.”* R3, NZ, Indigenous
Acceptability	*“Acceptability by intervention service providers is important for service provision, but needs to even more consider client acceptability first and that the intervention is delivered in an appropriate manner. There is too much history of clinical services “knowing best” in indigenous health.”* R4, NZ, Indigenous
Alignment	*“Stakeholder buy-in is important but it will be important to clarify which stakeholder opinions hold more weight when it comes to health equity!”* R3, NZ, Indigenous *“Alignment needs to focus on the community the intervention is being implemented in, not in external stakeholders.”* R7, US, Indigenous
Impact	*“Impact is not defined as a useful measurement for Indigenous communities in these listings of readiness levels. Focusing on providers and stakeholders is entirely inappropriate and likely to cause harm if the intended intervention is being considered for indigenous communities and what is good for indigenous communities can only be defined by them, not providers or stakeholders.”* R7, US, Indigenous *“Prioritize Indigenous stakeholders.”* R4, NZ, Indigenous

### New domains

General thematic analysis of questionnaire and interview responses led to the development of five new domains: Indigenous Data Sovereignty; Acceptability – Indigenous communities; Risk of research; Research team experience; Established partnership.

#### Acceptability – Indigenous communities

Several participants recognized the importance of Acceptability to ensure the intervention reflects providers’ priorities and is implemented as intended specifically in Indigenous communities. However, they raised the need to engage providers in preparatory work relating to health equity to ensure or increase acceptance and therefore the potential for intervention success, especially if intervention elements or implementation approaches differed from practices used by staff from dominant cultures in implementation sites.

*Health equity research findings can be confronting to many in the mainstream who don’t believe there is a problem.* (R3, NZ, Indigenous)

Most participants suggested broadening Acceptability to include the community in which the intervention will be examined, as without this acceptance the intervention is also likely to fail.

*[We need to think about] how we make research attractive to Indigenous communities”* (R7, US, Indigenous).

Participants felt Alignment was critical to Indigenous intervention development and implementation, like Acceptance. Many felt that the requirement for Indigenous stakeholders’ values and priorities needed to be explicitly stated. Participants mentioned the potential for stakeholders to hold competing priorities and some stated that Indigenous priorities need to be privileged above other stakeholders’. Although several participants recognised the need for some alignment between all stakeholders, including Indigenous stakeholders, they questioned what course of action to take when health systems or providers disagreed or did not value equity as a priority.

*Important question, but how are community needs balanced with stakeholder needs?* (R19, NZ, Indigenous)

#### Risk of research

Most respondents felt that understanding potential risks in Indigenous communities was essential for assessing readiness. In fact, some felt that researchers should assess risk first and not assess other domains or proceed with an ePCT if risk was unknown or there was potential for harm. Importantly, they described considering risk from the perspectives of both participants and the wider community, and not just risks associated with the intervention, but with the research process as a whole.

*What is deemed as a risk? What might not be a risk for non-Indigenous peoples might be a risk for Indigenous peoples. Is the intervention culturally appropriate? Could possibly consider the benefits for Indigenous peoples too. Thinking about collective risk of intervention not just individual risk*. (R20, NZ, Indigenous)

#### Research team experience

Several respondents felt that lack of evidence in Indigenous populations could be overcome by adapting interventions proven efficacious in other populations in partnership with Indigenous communities, without the need for further testing ahead of ePCTs. Some felt Evidence should consider the Indigenous practices in place and valued for decades or centuries, and not be limited to Western approaches to evidence. To undertake this however, it was identified that at least some members of the research team should have experience working with Indigenous communities to support these practices and that Indigenous researchers and communities should be part of the research team.

*[Indigenous communities] have to be part of the research team from the start, deciding the questions, methods and protocols.* (R19, NZ, Indigenous)

Respondents commented that such guidance accompanying RAPT-I would be particularly important for research teams with limited health equity experience; they felt that such researchers often want to do the right thing but lack the expertise to plan for equity.

#### Established partnership

Respondents discussed the importance of collaboration with Indigenous communities to assess each domain and facilitate culturally-appropriate modifications. They felt that the type and extent of preparatory work required prior to moving forward with an ePCT should be done by researchers and Indigenous communities together and that a modified RAPT could provide a useful framework for such planning and work. For example, some suggested modifying the domain to ensure the protocol be developed in partnership with Indigenous communities, explicitly consider health equity, and be written in culturally-appropriate and accessible language. Many emphasized the importance of engaging the Indigenous community to assess feasibility and recommended providing guidance to help researchers and communities identify all aspects of feasibility that should be assessed. Several participants also suggested identifying the communities’ opportunities and strengths which facilitated feasible implementation, rather than only shortcomings. Others noted that established partnerships would support the inclusion of outcome measures of most importance or relevance to Indigenous communities and that pre-work should ensure that planned outcomes are appropriate.

Importantly, respondents discussed the need to establish partnerships between researchers and Indigenous communities very early in the process.

*Partnership discussions should be part of the initial engagement.* (R7, US, Indigenous)

#### Indigenous Data Sovereignty

Interview participants highlighted the importance of Indigenous Data Sovereignty, with one respondent saying it was so important that it should be prioritized as the first domain. Respondents stated that Indigenous Data Sovereignty needed to be considered and discussed right from the outset and that these discussions were likely to be fundamental to partnership establishment and intervention implementation. Respondents felt that decisions that upheld Indigenous Data Sovereignty needed to be ongoing throughout the research process and therefore, that a shared understanding of the need for ongoing discussion was needed prior to e-PCT. Respondents also advised the framing of Indigenous Data Sovereignty guidance and scoring was important to demonstrate that research processes needed to ensure Indigenous rights to data sovereignty could be exercised.

*Indigenous communities will always have sovereignty over their data, but they may not have the infrastructure in place to exercise the sovereignty over their data.* (R5, US, Indigenous)

**Table 3:** RAPT-Indigenous (RAPT-I) - questions and scoring guidance for research with Indigenous communities

RAPT-I provides a framework for assessing research and intervention readiness prior to progressing with an ePCT. Areas assessed as low or medium readiness need to be reviewed and decisions made about whether these can be easily addressed, whether they can be addressed during the ePCT, or whether further preparatory work is required prior to progression to ePCT. It is intended that RAPT-I is used to assess readiness in collaboration between research teams and Indigenous communities. Assessment could be undertaken by the research team and Indigenous communities either separately or in collaboration. Application of RAPT-I requires critical assessment. It provides a framework by which reasons for scores can be justified and documented which, in addition to ePCT planning, has the potential to be useful in funding and ethics/institutional review board assessments of proposed ePCTs. For each domain, guidance has been added to support interpretation and scoring while the assessment is being undertaken. Domains would likely be scored low if no consideration has been made in that area. For example, the ‘Risk of Intervention’ domain would score low if no assessment of risk (formal or informal) had been undertaken. Low scores in some domains, for example impact and research partnership, may effectively serve as ‘stop’ criteria, whereby the research requires further development prior to continuation.			
**DOMAIN (modification)**	**Question and scoring criteria**		
**Implementation protocol** Question unchanged, scoring changed	**Original** ***Is the protocol sufficiently detailed to be replicated?***		
	**Proposed (unchanged)** ***Is the protocol sufficiently detailed to be replicated?***		
	**Guidance** To be sufficiently detailed, a protocol should consider equity at all stages of implementation. Understanding implementation requirements, and hence readiness, in Indigenous communities is ideally undertaken in collaboration *with* Indigenous communities. Inclusion in the research team of those with expertise in health equity is advantageous in designing for health equity. Replicability is a marker for a well-documented implementation process rather than a recommendation that interventions should be exactly replicated in different populations. At the readiness assessment stage, protocol does not need to be implemented, the protocol only needs to be available.		
	**Low**	**Medium**	**High**
	There is no protocol.	The protocol provides some documentation for implementation in the Indigenous communities, but some pathways are still uncertain/undocumented.	The protocol is well documented. All stages of implementation have been assessed to explore how the intervention will deliver equitable access, quality, and outcomes and these have been explicitly documented in the protocol.
**Evidence** Question changed, scoring changed	**Original** ***To what extent does the evidence base support the intervention’s efficacy?***		
	**Proposed** ***To what extent does the evidence base support the intervention’s efficacy in the Indigenous communities and setting(s) of interest?***		
	**Guidance** Evidence needs to be reviewed with respect to the Indigenous communities and setting(s) in which ePCT will be undertaken. When the Indigenous communities and/or setting vary significantly from those in which previous efficacy studies have been undertaken, thought needs to be given to whether prior feasibility studies should be undertaken to test modification of trial design or intervention. Efficacy is a measure of whether the intervention is beneficial under ideal and controlled circumstances.		
	**Low**	**Medium**	**High**
	There is no evidence of efficacy in Indigenous communities of interest.	Studies have demonstrated efficacy in the Indigenous communities of interest and setting but have either not been conducted using rigorous methods or have not been culturally appropriate. OR The intervention has been used in practice in the Indigenous communities but has not been formally evaluated.	Studies using rigorous, culturally appropriate methods have demonstrated efficacy in Indigenous communities and setting of interest.
**Risk** Question changed, scoring changed	**Original** ***Is it known how safe the intervention is?***		
	**Proposed** ***How safe is the intervention from a clinical and cultural perspective?***		
	**Guidance** Safety of the intervention should be scored from both clinical and cultural perspectives. Scoring of risk posed by the intervention should be undertaken in collaboration *with* the Indigenous communities and is likely to require an understanding of the historical, political and cultural contexts of the Indigenous communities. Indigenous community strengths which balance potential risks could be noted. Even if the intervention is scored as high readiness for this domain, processes to monitor for unanticipated harms should be in place.		
	**Low**	**Medium**	**High**
	The clinical and/or cultural risks (harms and discomforts) of the intervention are more than minimal (e.g., greater than ordinarily encountered in daily life).	Risk assessment of the intervention has not been completed, but the clinical and cultural risks are likely to be minimal.	Risk assessment of the intervention demonstrates that the clinical and cultural risks are minimal.
**Feasibility** Question unchanged, scoring changed	**Original** ***To what extent can the intervention be implemented under existing conditions?***		
	**Proposed (unchanged)** ***To what extent can the intervention be implemented under existing conditions?***		
	**Guidance** When scoring feasibility, the structural issues with resourcing should also be taken into consideration. Thought should be given to allowing for larger research budgets to account for potential underfunding/under-resourcing that already exists within the setting or Indigenous communities. Strengths and opportunities that exist within the Indigenous communities which facilitate feasible implementation should also be reviewed, in collaboration with Indigenous communities.		
	**Low**	**Medium**	**High**
	Necessary resources (e.g., staff, infrastructure, payment) are absent, or insufficient for implementation in the Indigenous communities.	Implementation in the Indigenous communities is possible with minor modifications to existing resources.	Implementation in the Indigenous communities is possible with existing resources.
**Measurement** Question unchanged, scoring changed	**Original** ***To what extent can the intervention’s outcomes be captured?***		
	**Proposed (unchanged)** ***To what extent can the intervention’s outcomes be collected?***		
	**Guidance** Some outcomes may be at the community level rather than individual level. This domain relates to the feasibility of *collecting* outcomes. Review of whether the measured outcomes are appropriate and relevant to the Indigenous communities is better considered within ‘Impact’.		
	**Low**	**Medium**	**High**
	Outcomes cannot be collected without major modifications to systems (e.g., clinical assessments, documentation, or electronic health records) or increases in staff time.	Outcomes for the Indigenous communities can be collected with minor modifications to systems or increases in staff time.	Outcomes for the Indigenous communities are already routinely collected and are complete.
**Cost** Question changed, scoring unchanged	**Original** ***How likely is the intervention to be economically viable?***		
	**Proposed** ***To what extent is the intervention economically viable in the Indigenous communities?***		
	**Guidance** Economic viability scoring requires that the costs and benefits of the intervention have been explored in terms of what holds value in Indigenous communities. It may require analysing the impact of the wider determinants of health on economic viability both in terms of intervention implementation costs and the potential for economic gain/loss. Review of structural factors that may affect implementation, such as chronic underfunding of Indigenous providers, should be considered and adjusted for in research budgets as appropriate.		
	**Low**	**Medium**	**High**
	Cost-benefit/cost-effectiveness analysis has not been completed (formally or informally) and it is unknown whether benefits outweigh costs.	Cost-benefit/cost-effectiveness analysis has not been completed, but benefits are likely to outweigh costs.	Cost-benefit/cost-effectiveness analysis demonstrates benefits outweigh costs.
**Acceptability – provider** Domain name changed, question unchanged, scoring unchanged	**Original** ***How willing are providers likely to be to adopt the intervention?***		
	**Proposed (unchanged)** ***How willing are providers likely to be to adopt the intervention?***		
	**Guidance** Perceptions of feasibility, need and value of the intervention may be affected by racism or biases. Where provider acceptability is low-medium, analysis of reasons for low acceptability is required to understand drivers of these perceptions, prior to moving forward to ePCT. Where the perceived lack of feasibility or need is rooted in racism or biases, assessment as to whether this can be overcome with support and/or education should be undertaken.		
	**Low**	**Medium**	**High**
	Acceptability is unknown or staff are unlikely to believe the intervention is feasible or needed.	Acceptability is unknown, but staff are likely to believe the intervention is feasible or needed.	Acceptability is known and staff believe the intervention is feasible and needed.
**Alignment** Question changed, scoring changed	**Original** ***To what extent does the intervention align with external stakeholders’ priorities?***		
	**Proposed** ***To what extent does the intervention align with stakeholders’, including the Indigenous communities’, priorities?***		
	**Guidance** When there are conflicts in priorities between different stakeholders, the priorities of the Indigenous communities should be upheld. There is the potential that the ‘competing’ priorities between various stakeholders can be managed and priorities of multiple stakeholders can also be addressed. Collaboration with stakeholders, including Indigenous communities, when planning the ePCT is likely to support this process and reduce these conflicts.		
	**Low**	**Medium**	**High**
	Stakeholders (policymakers, payors, advocates, Indigenous communities, and others) do not believe the intervention addresses a current or anticipated priority.	Some stakeholders, including Indigenous communities, believe the intervention addresses a priority.	Most or all stakeholders, including Indigenous communities, believe the intervention addresses a priority.
**Impact** Question unchanged, scoring changed	**Original** ***How useful will the intervention’s results be?***		
	**Proposed (unchanged)** ***How useful will the intervention’s results be?***		
	**Guidance** When scoring readiness for impact, equity-centred care and the policy and intervention benefits to Indigenous communities need to be considered, as well as the impact on other stakeholders. Indigenous community strengths that increase the potential for impact should be noted. Full assessment of the potential for impact should be undertaken in collaboration *with* Indigenous communities.		
	**Low**	**Medium**	**High**
	Providers and stakeholders (Indigenous communities, policymakers, payors, advocates, and others) do not, or are unlikely to, believe that the outcomes are useful (e.g., to inform clinical care or policy).	Some providers or stakeholders, including Indigenous communities, are likely to believe the outcomes are useful.	Most or all providers and stakeholders, including Indigenous communities, believe the outcomes are useful.
**NEW DOMAINS**	**Question and scoring criteria**		
**Indigenous Data Sovereignty**	**Proposed** ***To what extent do Indigenous communities hold sovereignty over research data?***		
	**Guidance** To protect the rights of Indigenous communities, it is essential that data sovereignty is discussed prior to ePCT. Indigenous Data Sovereignty recognises the right of Indigenous populations to control data from and about their communities, including what is collected, how data is interpreted and used, how and where the data is stored, and how and where the information is disseminated [45]. The proposed research should support Indigenous Data Sovereignty by recognising and protecting the rights of Indigenous communities.		
	**Low**	**Medium**	**High**
	Indigenous Data Sovereignty has not been considered in the context of the research project.	The research team and Indigenous communities have discussed data sovereignty, but not made any formal agreements.	The Indigenous communities has sovereignty over research data, with formal agreements specifying the extent and limitations of data access and use.
**Acceptability – Indigenous communities**	**Proposed** ***How willing are the Indigenous communities likely to be to adopt the intervention?***		
	**Guidance** Scoring of acceptability to Indigenous communities must take place in collaboration with Indigenous communities.		
	**Low**	**Medium**	**High**
	Indigenous communities believe the intervention is not feasible, acceptable, appropriate, or needed.	Acceptability is unknown, but Indigenous communities are likely to believe the intervention is feasible or appropriate or needed.	Acceptability is known and Indigenous communities believe the intervention is feasible, appropriate, and needed.
**Risk of research**	***How safe are the research and related processes for Indigenous communities?***		
	**Guidance** This domain specifically relates to research processes, as opposed to the previous domain regarding risk of the *intervention*. Even if an intervention is deemed safe, the research processes being used to test effectiveness could be potentially harmful. It is essential that this risk is assessed in collaboration with Indigenous communities. Thought needs to be given to both individual and collective/community risk and the historical, political and cultural contexts of Indigenous communities. Where areas of risk are identified, the research team and Indigenous communities should assess whether these risks are able to be easily mitigated through changes in the proposed implementation. Risk of research may be mitigated by the appropriate use of Indigenous research methods and methodologies.		
	**Low**	**Medium**	**High**
	Risk assessment has not been completed (formally or informally) and it is unknown whether the risks (harms and discomforts) of the research are more than minimal	Risk assessment of the research processes has not been completed, but the risks are likely to be minimal.	Risk assessment of the research processes demonstrates that the risks are minimal.
**Research team experience**	**Proposed** ***To what extent does the research team have the experience to undertake research with the Indigenous communities?***		
	**Guidance** Assessment of the appropriateness and skills of the research team conducting the research should be undertaken in collaboration with Indigenous communities. If previous engagement with the researcher(s) by Indigenous communities have been negative, it is likely that this domain will be scored ‘low’.		
	**Low**	**Medium**	**High**
	No one on the research team has appropriate experience working with Indigenous communities. The research team does not include any Indigenous researchers.	The members of the research team have some experience working with Indigenous communities. The research team has one team member from an Indigenous community.	Members from the population of interest are part of the research team. The research team as a whole has expertise working in partnership with the Indigenous communities of interest. The Indigenous communities have confidence in the ability of the research team to undertake with Indigenous communities. The research team has one or more members from the participating Indigenous community, or another Indigenous community.
**Established partnership**	**Proposed** ***To what extent has a partnership between researchers and Indigenous communities been established?***		
	**Guidance** Partnership requires equal power sharing with discussion and agreements, in this case between researchers and Indigenous communities, about how research will be undertaken, how rights will be protected, and how the research findings will be used. The CONSIDER statement [5] provides in-depth detail on aspects that should be considered for those less familiar with the development of authentic partnerships. Working in partnership will enable thorough readiness assessment using this tool.		
	**Low**	**Medium**	**High**
	There is no established partnership between the research team and Indigenous communities.	The research team and Indigenous communities have discussed a partnership but have not finalized and/or operationalized agreements OR the partnership does not have agreements and equal power sharing.	The research team and Indigenous communities have formalized and operationalized a partnership with equal power sharing.

## Discussion

Questionnaires and interviews with researchers conducting non-pharmacological dementia care interventions with Indigenous communities in NZ and the US resulted in recommendations to modify RAPT to explicitly incorporate considerations for pro-equity research in Indigenous communities. Recommendations for RAPT-I included new guidance for existing RAPT domains and the addition of new domains focused on Indigenous rights to culturally-safe research practices and to govern and control research processes. Participants also discussed how RAPT-I could guide researchers with limited experience with equity-focused research and emphasized the importance of assessing and modifying interventions in collaboration *with* Indigenous communities.

Others have previously raised the need for implementation science theory and methods to adequately incorporate health equity [32–34]. Without doing so, traditional implementation science will likely widen disparities, moving us further away from the goal of health equity [33]. Similar to our study findings, exploring and addressing power dynamics, working in partnership with the goal of developing sustainable models of care, and examination of wider structural systems that impact on interventions and their impacts have been deemed important [32]. As in our study, methods that facilitate and provide guidance on how to effectively design for equity when implementing an intervention have been identified as crucial [33, 34]. An example of how this is done in practice is provided by the National Institute of Aging IMPACT Collaboratory, which has produced guidance documents on ‘Best Practices for Integrating Health Equity into Embedded Pragmatic Clinical Trials for Dementia Care’ to step researchers through equity considerations at all stages of ePCT from community engagement and study design through implementation and analysis [35]. This type of tool, along with implementation frameworks addressing equity in Indigenous populations [11], could be used alongside RAPT-I, providing guidance on next steps if RAPT-I identifies low readiness in one or more of the domains.

Previous Canadian research investigating practices that support cultural safety in controlled trials with Indigenous peoples identified that effective communication and co-design between researchers and Indigenous communities and critical reflection in response to cultural ‘mistakes’ fostered success in research [36]. Indigenous peoples’ rights to control and power within research appeared to be recognised by participants who sought mechanisms within RAPT-I to protect and uphold these rights in implementation research. This included understanding the participation, and potential risk to communities, as a collective rather than solely as individuals, as well as recognising strengths and opportunities within communities. The CONSIDER statement [6] was developed to facilitate full and complete reporting of research that involves Indigenous peoples, however it also provides a framework through which to *plan* research that upholds Indigenous rights. Application of the CONSIDER statement would also be useful for planning for ePCT readiness in Indigenous communities.

Some of the concepts that are incorporated within RAPT-I domains have been previously described in pragmatic controlled trial literature with Indigenous populations. These include developing effective relationships which give power to Indigenous communities [36], Indigenous community endorsement of ePCT prior to initiation [37], relationships, assessing community and researcher readiness to commence the ePCT [22]. Previous work has identified ten principles of practice when undertaking health research with Indigenous Australians, although not specifically related to ePCT [38]. The adaption of interventions for ePCT with Indigenous communities has also been described, with methods for adaption including community involvement and focussing on strengths within Indigenous communities [39] and inclusion of culturally relevant values and materials [40, 41]. A recent scoping review identified that although participatory research approaches with Indigenous communities are needed for appropriate adaption, this is done with varying authenticity and success, and authors noted that clearer guidance was needed to facilitate improved practices [42]. Our research further builds on these works and brings together considerations relating to both intervention implementation and research processes in one tool for researchers and communities to access and guide them through an explicit ePCT readiness assessment process.

It is widely acknowledged that pragmatism of a clinical trial should be viewed on a continuum [18] and participants felt RAPT-I could provide a useful framework for researchers and Indigenous communities to critically and collaboratively evaluate readiness in Indigenous and equity focused contexts. Where there was low or medium readiness in some domains, participants felt that this would not necessarily prevent progression to ePCT, but that researchers and Indigenous communities should have collaborative discussions with decisions made about the preparatory work which could increase readiness. This may include small pilot or feasibility studies to better understand some aspects of the intervention and research processes.

Where research was not feasible due to structural factors such as chronic under resourcing as seen in other studies [43, 44], thought should be given to whether these could be corrected in the short-term. For example, those delivering the intervention could be resourced through research funding during the research contract, alongside researchers working with other stakeholders to advocate for and develop stable resourcing for sustainable service models in the long-term. This highlights the potential of researchers as advocates for structural change within health research resourcing. This includes a responsibility to monitor RAPT-I utilization to ensure it is used to strengthen research undertaken in and with Indigenous communities rather than impeding Indigenous progress.

RAPT was designed to help researchers make informed decisions about whether a particular intervention is ready to undergo real-world effectiveness testing and to identify areas that may need to be addressed prior to an ePCT. RAPT-I has the potential to also provide a useful framework for those charged with critically reviewing proposed pragmatic research, including funding and ethics review boards. Further study is warranted to pilot and refine RAPT-I within a broader context including non-dementia focused research and in Indigenous settings outside of NZ and the US. Further investigation to provide RAPT-I assessment exemplars, evaluate language accessibility, assess applicability in these additional settings and to explore how RAPT-I could be the basis for a broader health equity extension which would have applicability in the vast majority of ePCT readiness assessments would be beneficial.

### Strengths and limitations

A strength of this study was that the research team, and participants, had collective expertise in Indigenous health research, health equity, intervention science methodology and ePCT study design. Fewer participants than anticipated were required to reach data saturation in the online questionnaire. We only included researchers from the UA and NZ it is likely that this work can be progressed further by including other Indigenous populations and researchers. Participants were recruited from dementia-related studies only and widening the inclusion criteria may have led to more diverse discussion. Findings therefore may not be able to generalized for other study settings. Although participants with experience with research including Indigenous populations was sought, Indigenous health services research and development, or health equity more generally, may not have been their area of expertise.

## Conclusion

This study highlights the specific strategies to incorporate Indigenous health equity considerations into RAPT and offers RAPT-I as a proposed modified assessment. New domains have been proposed which advocate for the rights of Indigenous communities to be partners in research and maintain sovereignty over research data. RAPT-I provides a potential mechanism to increase the robustness of readiness assessment for ePCT by researchers and Indigenous communities.

### Electronic supplementary material

Below is the link to the electronic supplementary material.

Supplementary Material 1

## Data Availability

Data is not available as use by third parties was not granted in the ethics process.

## Abbreviations

ePCT: Embedded pragmatic randomized, controlled trials
NZ: New Zealand
RAPT: Readiness Assessment for Pragmatic Trials
RAPT-I: Readiness Assessment for Pragmatic Trials – Indigenous
US: United States of America
