# Supplementary material for: Modifications of the readiness assessment for pragmatic trials tool for appropriate use with Indigenous populations
Source: BMC Med Res Methodol. 2024 May 31;24:121. doi: 10.1186/s12874-024-02244-z (PMC11140978; doi:10.1186/s12874-024-02244-z)
Supplement: Supplementary file 1 — Supplementary Material 1 [file 12874_2024_2244_MOESM1_ESM.docx]

**Demographics**

1. What ethnic group do you belong to? You can name as many as you like.

Māori

Native Hawaiian

Native American or Alaskan Native

White or Caucasian or NZ European

Black or African American

Hispanic or Latino

Samoan

Cook Islands Māori

Tongan

Niuean

Chinese

Indian

Other:__________

2. Which country do you live in?

3. Please tick the type/s of research experience you have. Tick all that apply

Researcher

Principal investigator

Site lead

Qualitative research

Quantitative research

Health services research

Health equity research

Community member

Other

4. How many years’ experience do you have as a researcher in general?

5. How many years’ experience do you have specifically in Indigenous research?

6. How many years’ experience do you have in older adult and/or dementia-related research?

7. What is your highest university qualification?

Not applicable

Bachelors/undergraduate

Post-graduate diploma

Masters

PhD

Other doctorate

**Questions relating to RAPT domains**

**Introduction and instructions**

When developing and researching health interventions, it is important to understand the real-world benefits and risks. Testing interventions in a pragmatic randomized controlled trial (PCT) can help with this. A PCT requires a lot of resource and, if not planned appropriately, may also lead to false conclusions. To help with efficient use of resources and high quality PCT study design, initial research should have been done to understand whether an intervention is ‘ready’ to test in a PCT. The Readiness Assessment for Pragmatic Trials (RAPT) tool has been developed to help with this and it consists of nine domains, with researchers asked to score foreach domain to provide an overview of intervention ‘readiness’ for PCT. The RAPT tool was developed within a US National Institute of Ageing (NIA) funded Program specifically related to nonpharmacological dementia interventions.

To develop health interventions which have the potential to achieve equitable outcomes, it is important to design for equity. In other words, the goal of equitable outcomes needs to be actively planned for, and this includes in the development of interventions. The current RAPT tool does not explicitly assess intervention ‘readiness for PCT’ from a health equity perspective. In this questionnaire you be provided with a description of each of the current RAPT domains, including the scoring guidance. You will be asked to review the appropriateness of each of the current RAPT domains in the setting of dementia interventions in Indigenous populations.

**When thinking about developing dementia interventions and testing them in Indigenous communities, please rate the appropriateness of each domain listed in the following questions**. It may help to reflect on the research you have previously been involved in.

8.


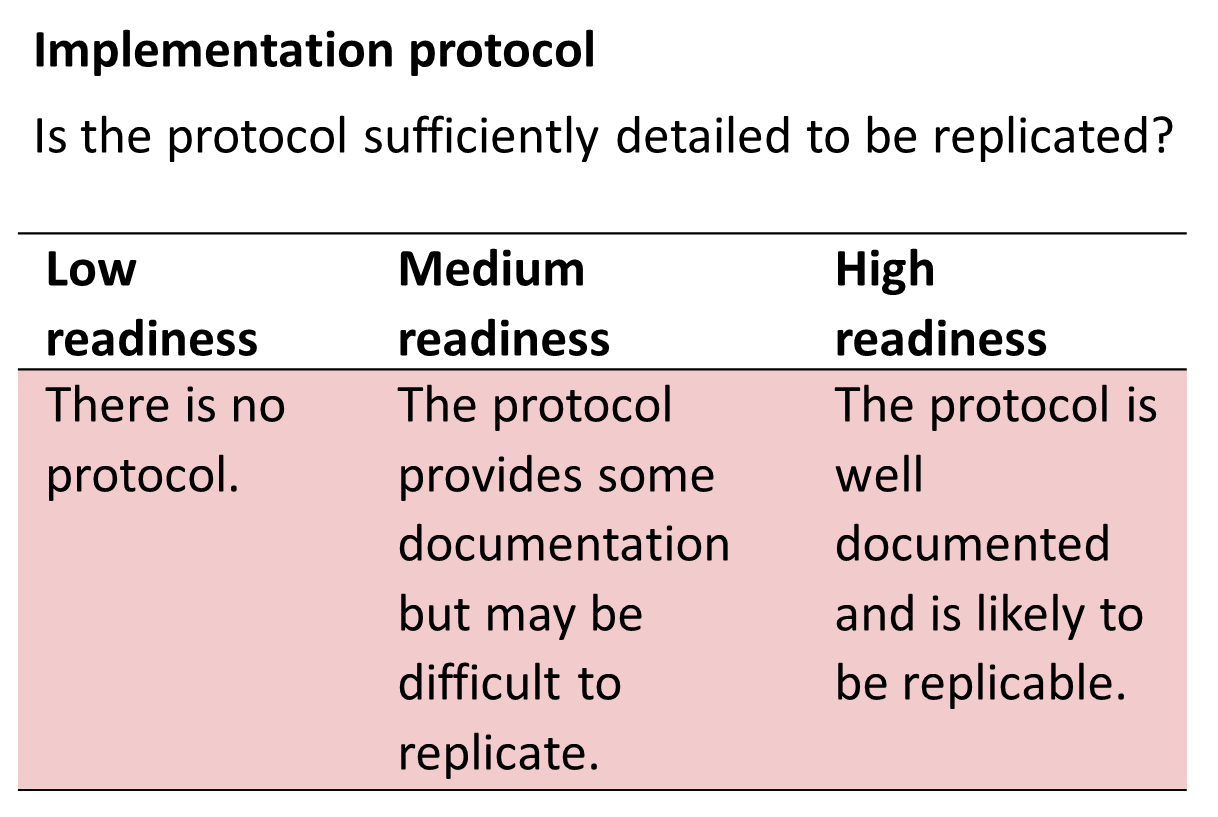


a. How appropriate is this domain?

Appropriate

Slightly appropriate

Slightly inappropriate

Inappropriate

b. Why did you give this answer?

c. To adequately incorporate health equity, should this domain:

Remain unchanged

Have additions made

Have aspects removed

Be completely rewritten

Be removed

d. If you responded above that some changes are required, please comment with the changes that are needed: [free text]

9.


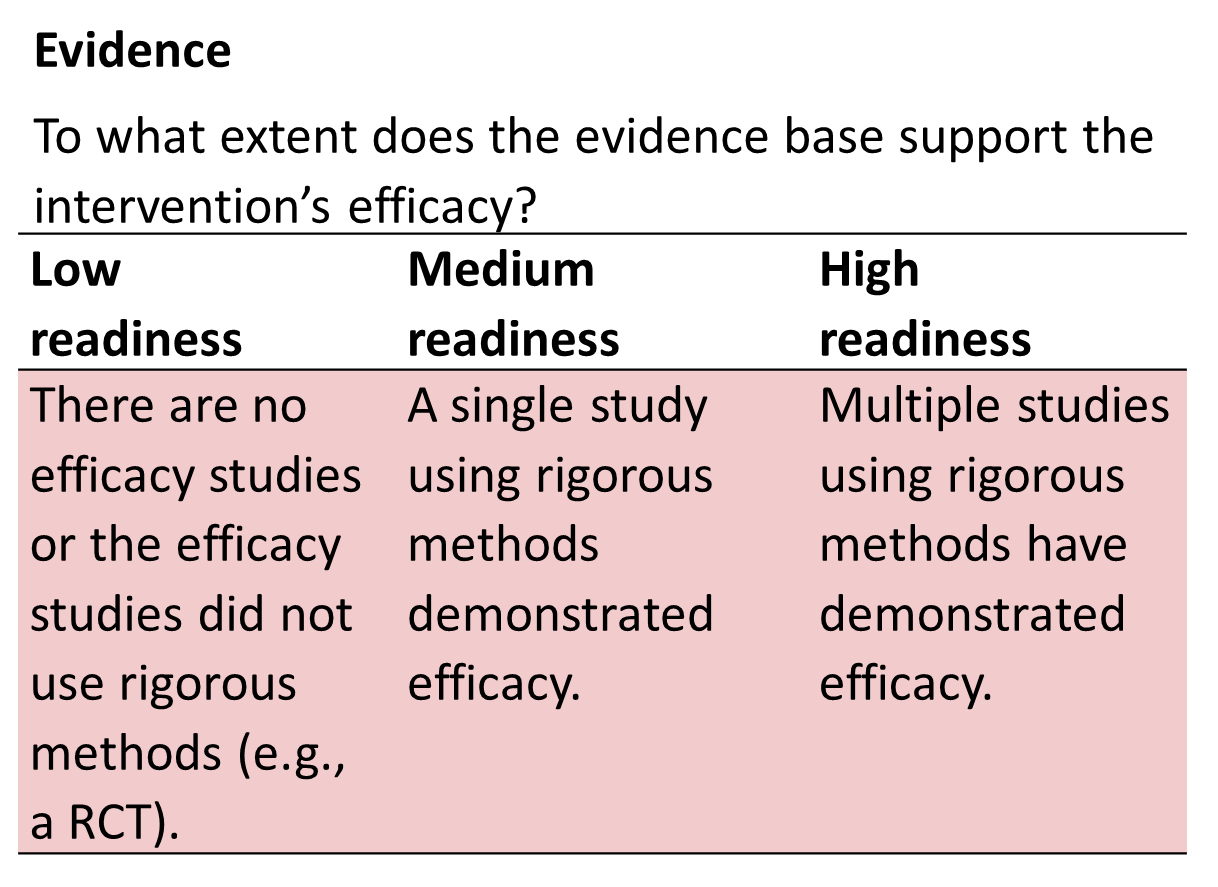


a. How appropriate is this domain?

Appropriate

Slightly appropriate

Slightly inappropriate

Inappropriate

b. Why did you give this answer?

c. To adequately incorporate health equity, should this domain:

Remain unchanged

Have additions made

Have aspects removed

Be completely rewritten

Be removed

d. If you responded above that some changes are required, please comment with the changes that are needed: [free text]

10.


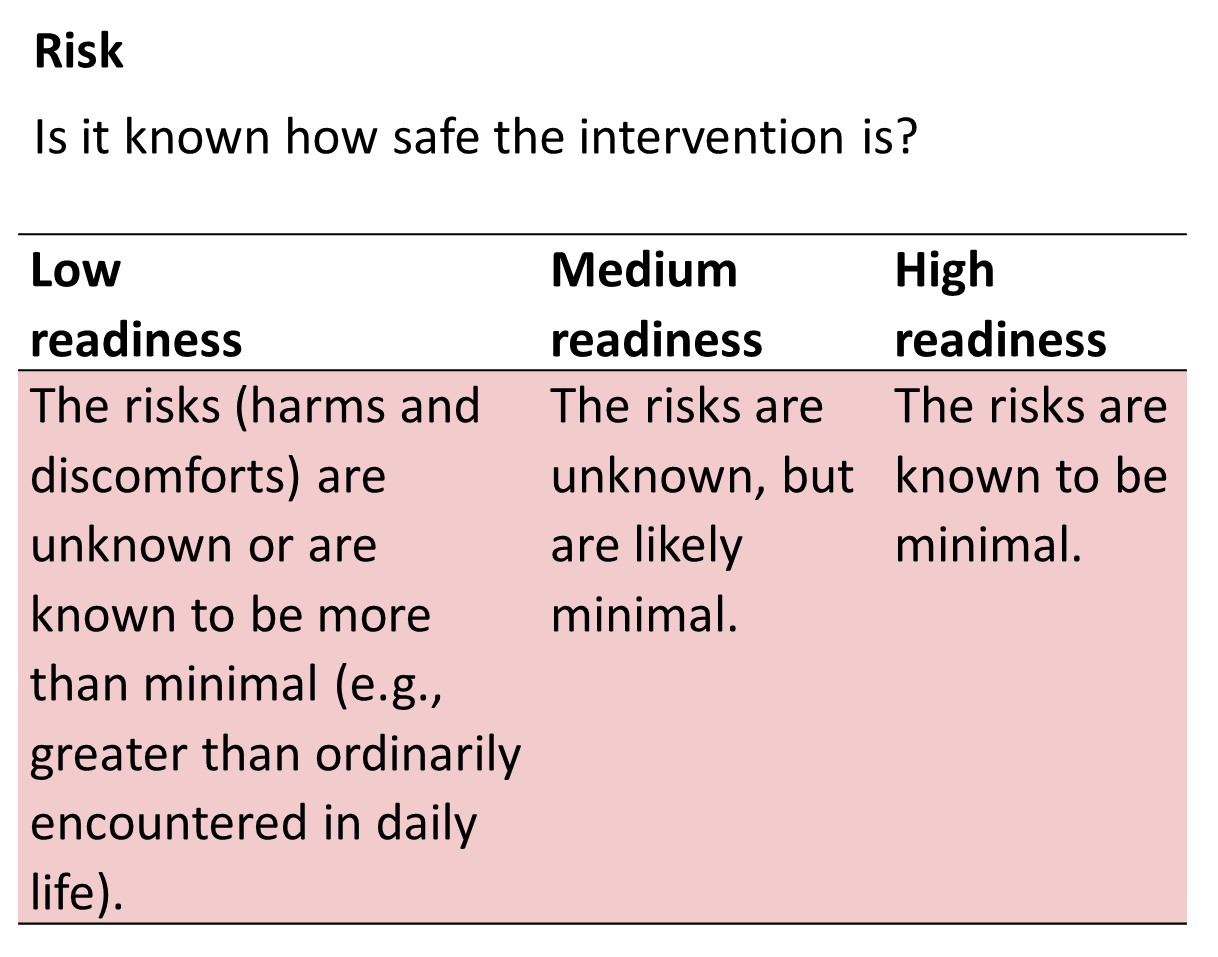


a. How appropriate is this domain?

Appropriate

Slightly appropriate

Slightly inappropriate

Inappropriate

b. Why did you give this answer?

c. To adequately incorporate health equity, should this domain:

Remain unchanged

Have additions made

Have aspects removed

Be completely rewritten

Be removed

d. If you responded above that some changes are required, please comment with the changes that are needed: [free text]

11.


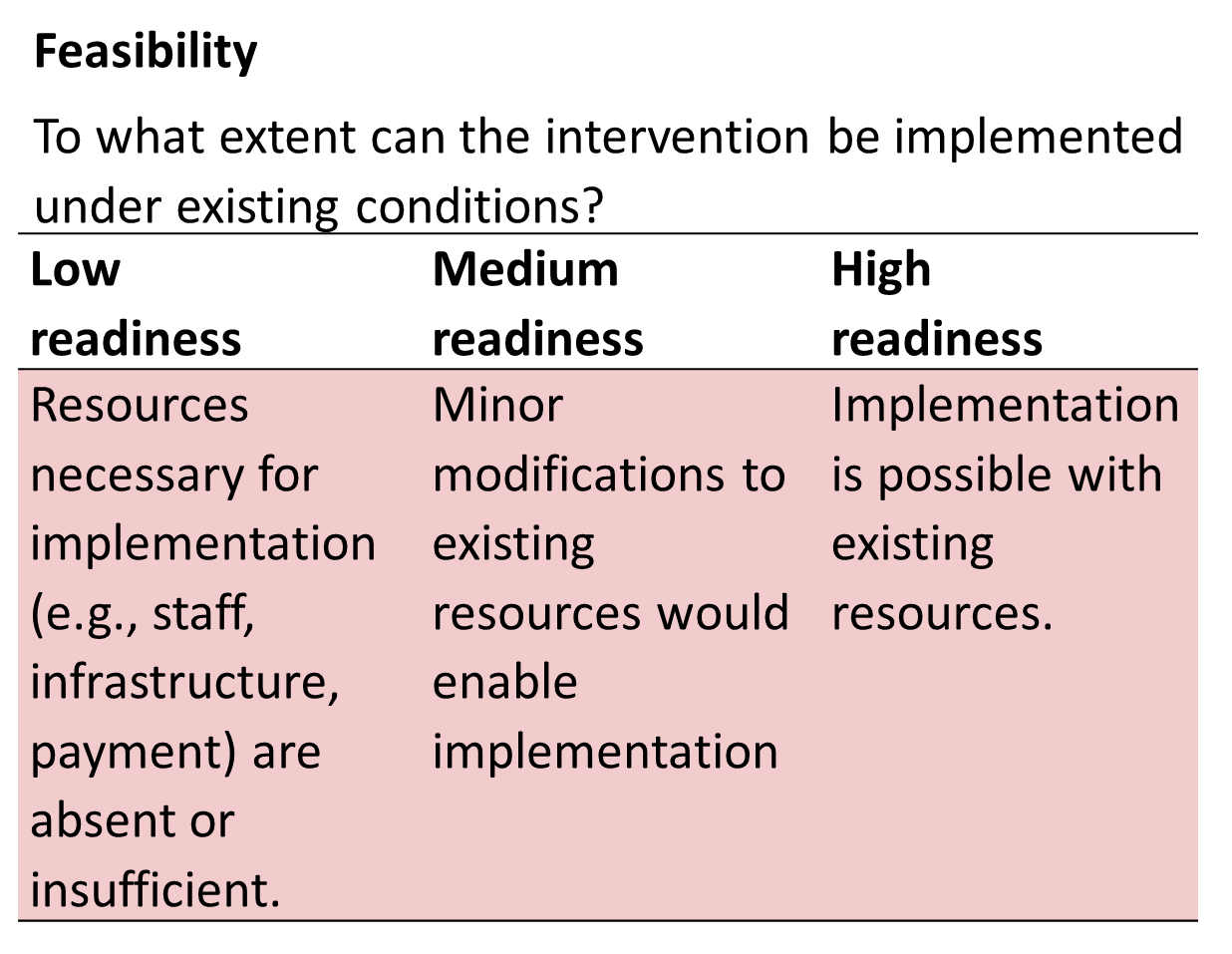


a. How appropriate is this domain?

Appropriate

Slightly appropriate

Slightly inappropriate

Inappropriate

b. Why did you give this answer?

c. To adequately incorporate health equity, should this domain:

Remain unchanged

Have additions made

Have aspects removed

Be completely rewritten

Be removed

d. If you responded above that some changes are required, please comment with the changes that are needed: [free text]

12.


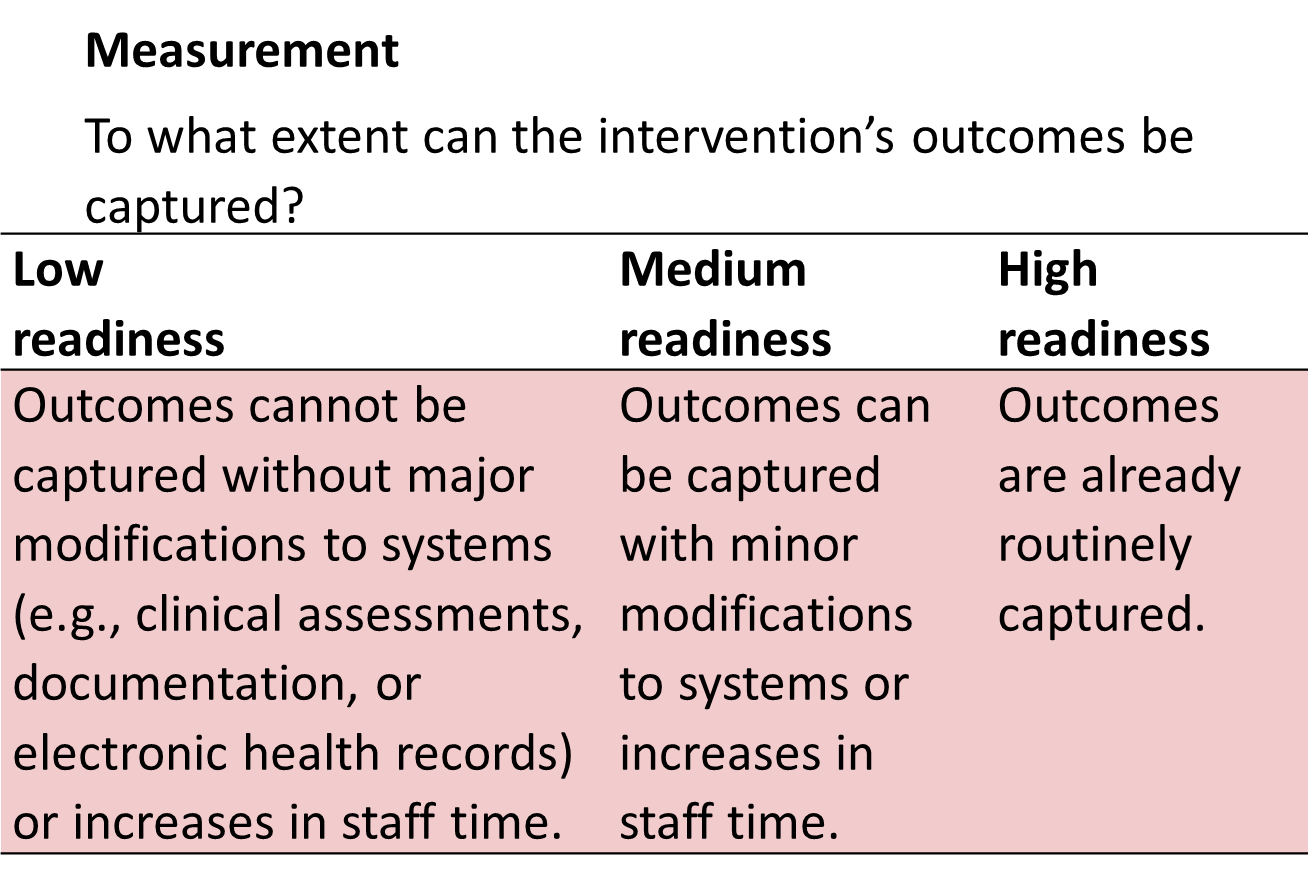


a. How appropriate is this domain?

Appropriate

Slightly appropriate

Slightly inappropriate

Inappropriate

b. Why did you give this answer?

c. To adequately incorporate health equity, should this domain:

Remain unchanged

Have additions made

Have aspects removed

Be completely rewritten

Be removed

d. If you responded above that some changes are required, please comment with the changes that are needed: [free text]

13.


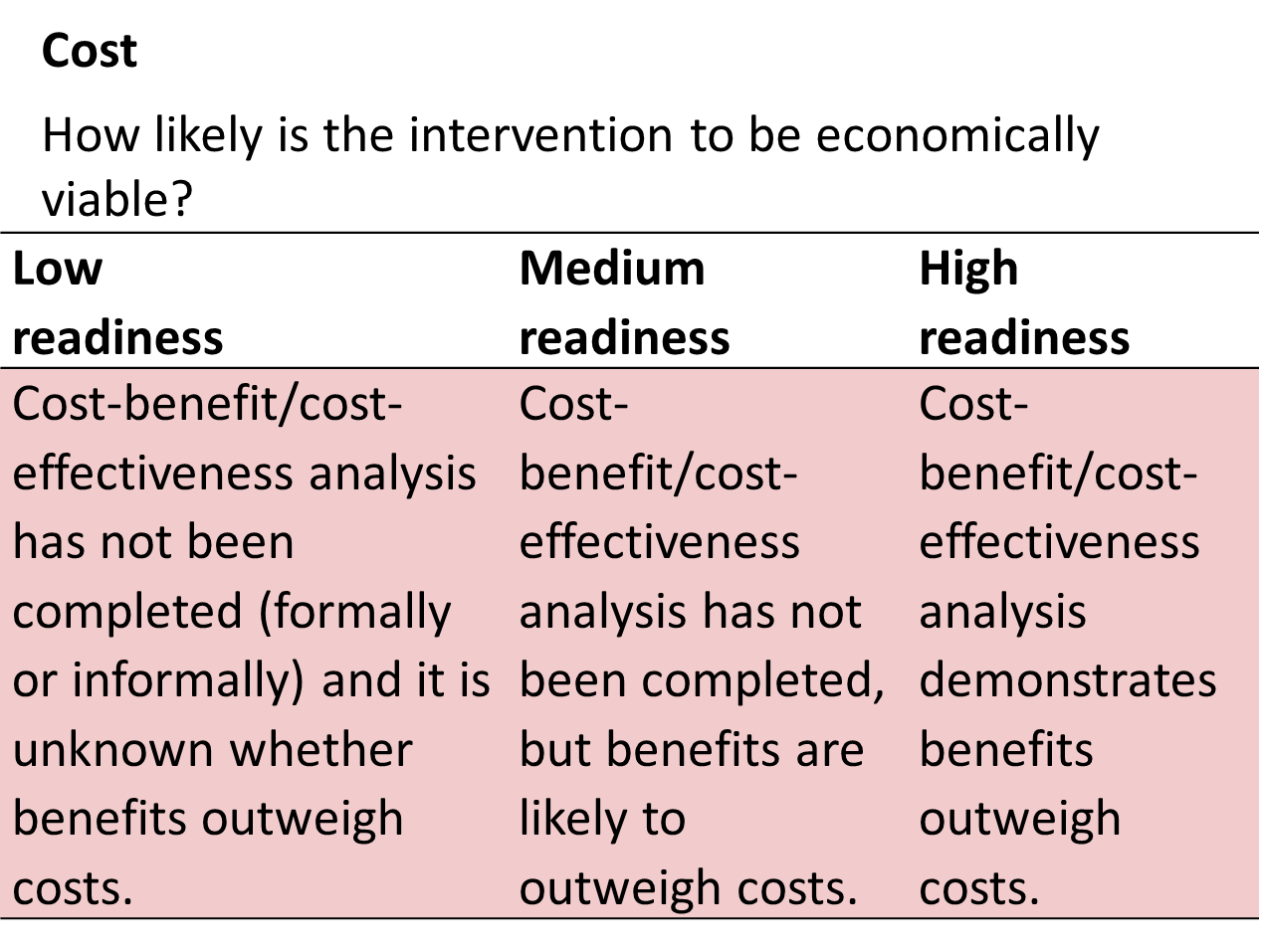


a. How appropriate is this domain?

Appropriate

Slightly appropriate

Slightly inappropriate

Inappropriate

b. Why did you give this answer?

c. To adequately incorporate health equity, should this domain:

Remain unchanged

Have additions made

Have aspects removed

Be completely rewritten

Be removed

d. If you responded above that some changes are required, please comment with the changes that are needed: [free text]

14.


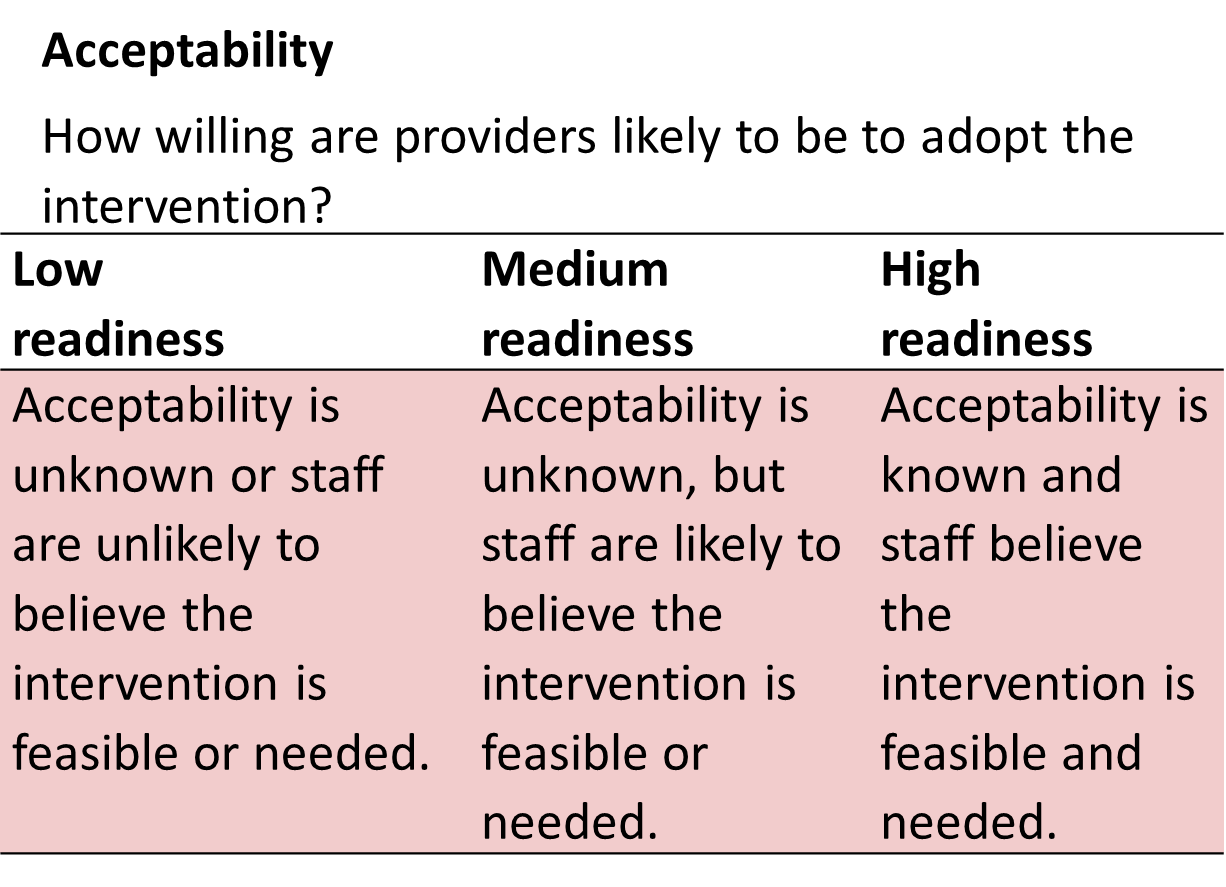


a. How appropriate is this domain?

Appropriate

Slightly appropriate

Slightly inappropriate

Inappropriate

b. Why did you give this answer?

c. To adequately incorporate health equity, should this domain:

Remain unchanged

Have additions made

Have aspects removed

Be completely rewritten

Be removed

d. If you responded above that some changes are required, please comment with the changes that are needed: [free text]

15.


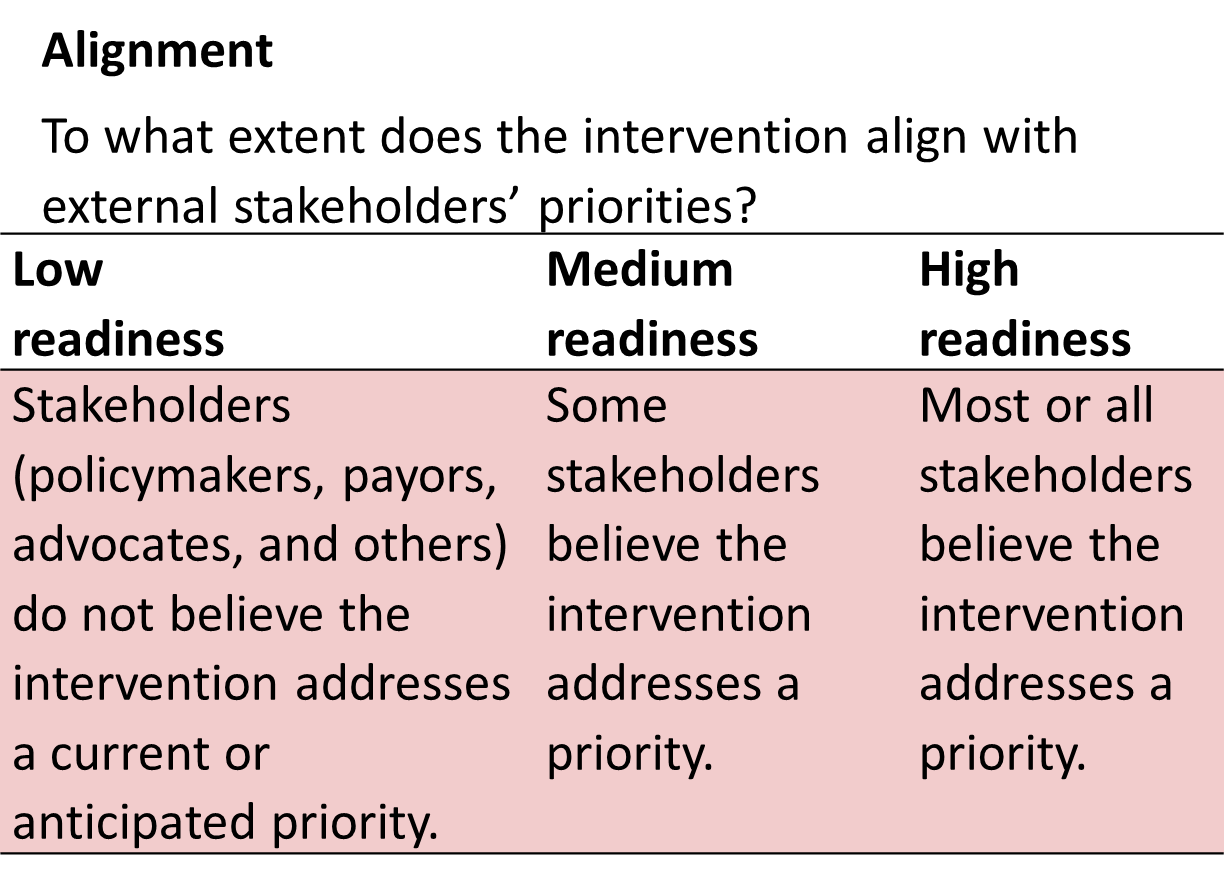


a. How appropriate is this domain?

Appropriate

Slightly appropriate

Slightly inappropriate

Inappropriate

b. Why did you give this answer?

c. To adequately incorporate health equity, should this domain:

Remain unchanged

Have additions made

Have aspects removed

Be completely rewritten

Be removed

d. If you responded above that some changes are required, please comment with the changes that are needed: [free text]

16.


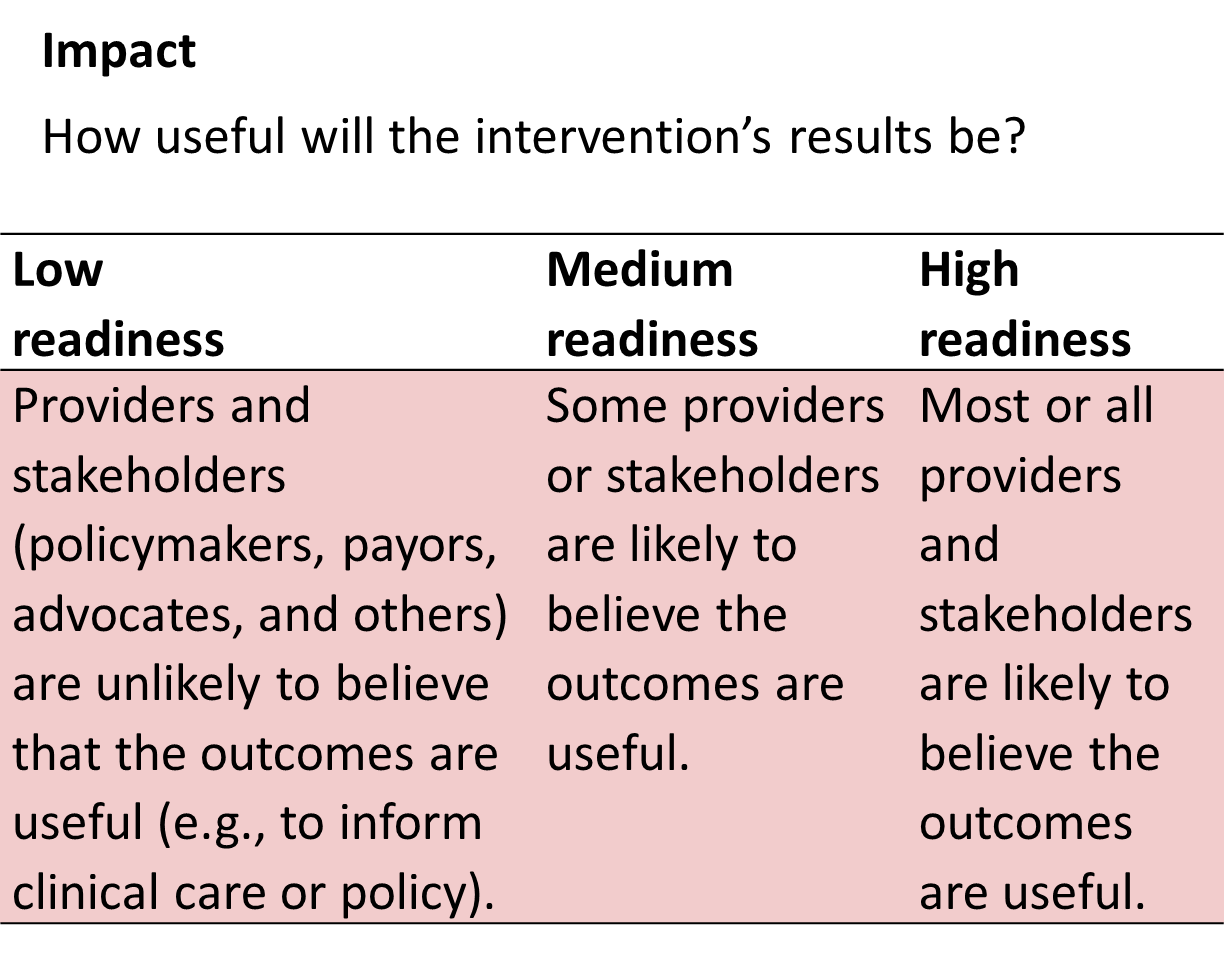


a. How appropriate is this domain?

Appropriate

Slightly appropriate

Slightly inappropriate

Inappropriate

b. Why did you give this answer?

c. To adequately incorporate health equity, should this domain:

Remain unchanged

Have additions made

Have aspects removed

Be completely rewritten

Be removed

d. If you responded above that some changes are required, please comment with the changes that are needed: [free text]

**RAPT modifications**

17. Are there any domains that should be added to the current RAPT tool?

Yes

No

If yes, which domains should be added? [Free text]

18. Would you use the RAPT tool in its current form to assess intervention readiness in the setting that you work?

Yes

No

Comment (optional): [Free text]

19. Are there any risks in using the RAPT tool in its current form that you have not raised elsewhere?

[Free text]

20. Would you use a version of the RAPT tool that had been modified to assess intervention readiness from a health equity perspective?

Yes

No

Comment (optional): [Free text]

21. Do you have anything else you would like to share?

[Free text]

**Legend**

Scoring guidance
